# Guideline-adherence in the treatment of symptomatic urolithiasis in children and adolescents in southwestern Germany

**DOI:** 10.1186/s12894-020-00643-0

**Published:** 2020-06-26

**Authors:** Felix Blasl-Kling, Simone Katrin Dold, Jan-Thorsten Klein, Gamal Anton Wakileh, Ulrich Humke, Anne-Karoline Ebert

**Affiliations:** 1 Department of Urology, Katharinenhospital, Kriegsbergstr. 60, 70174 Stuttgart, Germany; 2 Department of Pediatrics and Adolescent Medicine, Medical University Ulm, Eytthstr. 24, 89075 Ulm, Germany; 3 Department of Urology and Paediatric Urology, Medical University Ulm, Albert-Einstein-Allee 23, 89081 Ulm, Germany

## Abstract

**Background:**

Approximately 1% of urolithiasis cases in Germany affect children. Interdisciplinary groups have agreed on national and international guidelines for children to recommend appropriate treatment pathways. The aim of this retrospective and preliminary study is to analyze whether adherence to current guidelines for pediatric stone disease in southwestern Germany is feasible.

**Methods:**

During 2014 to 2017 24 children and adolescents (nine female, 15 male, median age 9.7 years), were treated for symptomatic urolithiasis in our institutions. We retrospectively collected clinical and operative courses. Clinical pathways were compared to previous guideline recommendations of the EAU 2014 and the German S2k guideline 2015.

**Results:**

17 of the 24 patients were treated according to guideline recommendations (71%). Non-adherency was based on parental decisions in two and technical/medical considerations in five cases. In 11 children (45.8%) secondary or adjunctive treatments were necessary, in three of the seven non-adherently treated (43%) and in eight of the 17 adherently treated children (47%).

**Conclusion:**

Our daily treatment approach seems to comply well with current pediatric stone guidelines. Nevertheless, guideline-non-adherent decision making emphasizes their strength and limitations, as specific clinical situations in children may require an individual treatment plan, as non-predictable conditions may occur.

## Background

Despite being a rare condition in children, accounting for about 1% of the whole stone population, urolithiasis is a wide-spread disease in developed countries with an increasing prevalence of about 5% in Germany and up to 10% in the US [1–3]. Depending on the age at presentation, general work-up in urolithiasis consists mainly but not exclusively of laboratory studies and stone visualization via ultrasound or X-ray, to further determine proper treatment pathways. Basing their recommendations on current literature, expert panels of different countries have condensed treatment pathways into various national and international guidelines to further improve treatment efficiency and lower complication and morbidity rate for the affected patients. For pediatric patients the EAU guidelines and the German S2k guidelines are available [4, 5]. Children are predestined high-risk stone patients due to a high rate of metabolic and anatomical abnormalities with a higher probability of stone recurrences. Following necessary repeated invasive stone treatment there is an increasing risk of long-term complications [6]. Urolithiasis in childhood itself and its high recurrence rate of about 50% have a major impact on daily life and the future kidney function for the affected pediatric patients [3]. These facts are reflected in the pediatric sections of the above mentioned guidelines with specific pediatric treatment recommendations [4, 5]. In this preliminary retrospective study we analyzed the clinical course and decision making in 24 cases of pediatric patients in two institutions in southwestern Germany, to further evaluate whether treatment guidelines in pediatric stone disease are feasible to follow in a complex patient cohort.

## Methods

This study retrospectively reviewed the clinical records of 24 consecutive children and adolescents diagnosed and clinically surveyed in Ulm University Hospital and Katharinen Hospital Stuttgart between 2014 and 2017. As the focus of our trial was on the guideline-adherence of a realized treatment plan and potential secondary interventions and complications, no patients were excluded. The anonymized patient files were analyzed in a standardized manner and epidemiological data such as age, gender, comorbidities and the following clinical course were collected. All included patients were followed at least up to the point of a stone free status as a consequence of our procedures. Due to the small patient population statistical analysis mainly used descriptive methods, including counts, means and ranges. Fisher’s exact test and the t-test were used to calculate differences in the rate of secondary procedures in the different groups of guideline-adherent and guideline not-adherent cases. Statistical significance was defined by *p* < 0.05. Analyses were performed by the statistics software SAS©, version 9.4 (SAS Institute Inc., Cary, N.C., USA).

## Results

The patient cohort consisted of 15 male and 9 female patients with a median age at presentation of 9.7 years, ranging from 3 months to 18 years (Table 1). The follow up at the Department of Pediatric Urology and Pediatric Nephrology was median 1.6 years, ranging from 3 months to 3 years. During the follow-up period five children turned 18 years. Two of them were lost to follow-up as adults; the other three were regularly seen by their adult urologists. In total four of 24 children had an underlying metabolic disease. Two children were diagnosed with cystinuria, another one with primary hyperoxaluria Typ II and one was identified with uric acid stones.

**Table 1:** Children with symptomatic urolithiasis treated in both institutes in a period between 2014 to 2017

N	Comorbidities	Age-range at presentation (^a^)	Affected side (L/R)	Stone position, size, density, hydronephrosis, underlying metabolic condition	Primary Treatment	Guideline Recommendation	Secondary Treatment
1	Osteogenesis imperfecta	adolescent	L	distal ureter, 3 stones: 3, 4, 6 mm, 50 HU, II° HN	conservative	conservative	0
2	type 2 diabetes, adiposity, hypothyreosis	adolescent	R	renal pelvis: 17 mm, 350 HU	SWL	SWL	1 (SWL)
3	premature infant, 26 + 2 week of gestation, twin I	toddler	R	renal pelvis: 12 mm, III° HN	presenting SWL	SWL	1 (SWL)
4	adiposity	adolescent	L	ureteral ostium: 2 mm	conservative	conservative	0
5	–	school-age	L	lower calix: 7 mm, III° HN, uric acid stones	chemolitholysis	chemolitholysis	1 (ureteral stent)
6	–	adolescent	L	ureteral ostium: 3 mm, 740 HU, II° HN	conservative	conservative	0
7	concurrent UPJO	toddler	L	stag-horn calculus: 20 mm, II° HN, struvit stone	Pyelolithotomy plus simultaneous pyeloplasty	PCNL	0
8	–	adolescent	L	distal ureter: 7 mm, II° HN	conservative	conservative	0
9	asthma	school-age	R	distal Ureter: 8 mm, II° HN	URS	URS	0
10	indeterminate colitis	adolescent	L	distal ureter: 5 mm, I° HN, rupture of renal fornix	prestenting flexible URS	URS	0
11	asthma	adolescent	L	proximal ureter: 5 mm, 210 HU, I° HN	conservative	SWL	1 (URS)
12	lactose intolerance	adolescent	L	proximal ureter: 3 mm, 460 HU, II° HN	SWL	SWL	0
13	iron deficiency anemia	toddler	L	renal pelvis: 8 mm, III° HN, type II primary hyperoxaluria	SWL	SWL	2 (URS)
14	–	infant	L	lower and middle calix: 6 mm each, cystinuria	prestenting flexible URS	URS	1 (URS)
15	depression, suspected developmental personality disorder	adolescent	L	proximal ureter: 4 mm, II° HN	conservative	SWL	0
16	major beta-thalassemia, S.p. bone marrow- and umbilical cord transplantation 12/2012	adolescent	R	distal ureter: 6 mm, II° HN	SWL	URS	0
17	–	school-age	R	ureteral ostium: 3 mm	conservative	conservative	0
18	right ureteral duplication, left dysplastic kidney	toddler	R	lower calix: 8 mm	prestenting flexible URS	SWL	0
19	–	school-age	L	lower calix: 10 mm, middle and upper calyxes: 5 mm each, III° HN	prestenting SWL	SWL	0
20	premature infant, 24 week of gestation short bowel syndrome	pre-school	R and L	R lower calix: 9 mm L renal pelvis: 20 mm, IV° HN (MAG3 scintigraphy: split function 89%: 11% R: L)	R presenting, URS L pyelolithotomy	R SWL L PCNL	2 (R SWL, L nephrectomy planned, split function 0%)
21	–	pre-school	L	renal pelvis: 15 mm, II° HN	prestenting SWL	SWL	2 (SWL, URS)
22	–	school-age	R	renal pelvis: 18 mm, II° HN	presenting SWL	SWL	4 (2 x SWL, 2 x URS)
23	–	school-age	L	renal pelvis: 4 mm lower calix 2 stones, 5 mm each cystinuria	SWL	URS	1 (SWL)
24	–	toddler	L	renal pelvis: 16 mm, II° HN	prestenting SWL	SWL	1 (SWL)

*HU* Hounsfield units, *HN* Hydronephrosis, *URS* Ureterorenoscopy, *SWL* Shock-wave lithotripsy, *PCNL* Percutaneous nephrolithotomy, *L/R* L left, R Right, *UPJO* Ureteropelvic junction obstruction, ^a^age-ranges at presentation: infant < 1 years, toddler 1–3 years, pre-school children 3–6 years, school-age children 6–14 years, adolescents 14–18 years

### Diagnostic procedures

All 24 children received ultrasound imaging at their first presentation and in each consecutive follow-up situation. Five of 24 children, in all cases children above the age of 14 years (median 16,4 years), received a low-dose CT-scan at their first presentation to further clarify stone localization. Stone size, location and density were collected in (Table 1) as well as the degree of hydronephrosis.

### Conservative management

Eight children (five male, three female, median age 14.75 years) were treated conservatively. Five of them received an off-label medical expulsion therapy (MET) with the alpha blocker tamsulosin in combination with ibuprofen or metamizole. In two patients only ibuprofen or metamizole was used. Five of the non-surgically treated patients had distal ureteral stones (median size 4 mm, min. 2 mm, max. 7 mm). Two male children had proximal ureteral stones (4 and 5 mm in size). In six cases the stones were successfully passed spontaneously without the need for adjunctive or secondary treatment. One boy with a 5 mm proximal ureteral stone needed a primary ureterorenoscopy (URS) to extract the stone completely after failure to pass it spontaneously. A 10 years old male patient with previously diagnosed uric acid stones could be treated with chemolitholysis for his 11 mm lower calix stone. Nevertheless, he later needed a double-J ureteral stent (DJ)-insertion to successfully pass all stone fragments over a period of 4 months.

### Shock-wave lithotripsy (SWL)

The most common invasive treatment method was shock-wave lithotripsy (SWL) using ultrasound adjustment. SWL was applied to one third of the whole group (*n* = 10) (Table 1). In six of these cases (three male, three female) a single renal pelvis stone of a size of 8 to 18 mm (median 14.3 mm) was treated. All six children needed a secondary operative intervention. Four of them (median stone size 13.25 mm) had an additional SWL session and two more with larger stones (median stone size 16.5 mm) a secondary SWL and an additional URS thereafter. One girl with cystinuria presented with three 4-5 mm renal pelvis and lower calix stones. She needed two SWL sessions to achieve a stone-free status. One seven years old female with a 10 mm lower calix stone and two males with ureteral stones (17 years old with a 3 mm proximal and 15 years old with a 6 mm distal stone) were successfully treated with one SWL session.

### Endourological treatment

Endourological treatment as a primary approach was scheduled for five children (four males at the age of 6, 2, 0.25 and 6 years, and one female at the age of 14 years). One of them, a 6 year old boy with an 8 mm distal ureteral stone had a successful primary URS without pre-stenting. The other four children needed a ureteral stent before URS treatment. In a female with a 5 mm distal ureteral stone a ureteral stent was inserted after a renal fornix rupture, later a semirigid ureteroscopy was done. In preparation for a flexible intrarenal URS three other children, all male, were pre-stented: one patient with two stones, situated in the lower and middle calix, 6 mm in size each, another one with an 8 mm lower calix stone of a duplicated system and one patient with a 9 mm right lower calix stone. The last patient had a 20 mm staghorn calculus in his contralateral malfunctioning left kidney (initially 11% partial function), which was treated by open-surgical pyelolithotomy according to the parental wish. He needed SWL on residual stones in his right kidney, while his left kidney did not show any function after successful stone therapy.

### Open stone therapy

Two of the 24 children were treated with primary open surgical technique. Additionally to the boy mentioned above, a 3 years old male underwent successful open-surgical pyelolithotomy and UPJ-reconstruction on a 20 mm struvit staghorn calculus in his left kidney due to a concurrent ureteropelvic junction obstruction.

### Guideline adherence

17 of the included 24 patients were treated adherently to the 2015 German S2k-guidelines and the 2014 EAU guidelines. In the seven remaining individuals the treatment plan was developed individually. In two cases with proximal ureteral stones the parents refused consent to a primary SWL treatment. Both children therefore primarily underwent a conservative approach, which was successful in one case but lead to a secondary URS in the other case. In two patients stone size, kidney function, anatomy and parental wishes lead to an open surgical approach instead of a percutaneous treatment (PCNL). A ureteral duplication in a further case argued for an endourological approach instead of the recommended SWL treatment. The sixth child not treated guideline-conform had a SWL on a distal ureteral stone instead of an URS, as this 15 years old boy had already needed a bone-marrow transplantation due to his thalassemia and therefore anesthesiological risks were tried to be minimized. In the seventh case, a 10 years old female diagnosed with Cystinuria short after birth, was treated with SWL on multiple stones in her kidney. Besides urine alkalization, she had not needed any intervention for stones so far, as she had been able to pass all stones spontaneously. In her case the individual approach led to sedation during SWL instead of a general anesthesia in case of an endourological treatment. Stone free status was achieved after two sessions of SWL.

In summary 11 of the children needed secondary or adjunctive treatment (Table 1). The proportion of additional procedures was almost similar in the guideline adherently treated (eight of 17) and the guideline non-adherently treated children (three of seven). SWL as a primary treatment method resulted in 8 consecutive interventions (seven guideline-adherently and one guideline-non-adherently treated). 4 of these needed a single additional SWL session to become stone-free. The other four children in this group needed two more SWL sessions or an additional URS. There was only one complication documented during primary and secondary treatment. This was a grade I complication according to the Clavien and Dindo classification; one three month old boy post-operatively presented with fever and needed antipyretic therapy after URS treatment; no urinary tract infection or dilatation of the kidney were detected.

## Discussion

Medical guidelines in an adult population are usually based on structured, at best randomized, controlled, large-scale investigations, which can easily and with a high degree of evidence be condensed into diagnostic and therapeutic recommendations. With regard to the rarity of pediatric stone disease and the difficulty to conduct structured large center trials in pediatric patients, the recommendations of the current guidelines are mainly based on case reports, small cohorts or single-center experiences and provide only a low level of evidence. Therefore the question arises whether these recommendations are practical, feasible and reliable for the patient’s best care. From a clinical point of view individual treatment courses for patients with pediatric stone disease often seem very complex, featuring difficult anatomical conditions and metabolic comorbidities. Therefore, guideline recommendations are limited and individual deviations may be required. Additionally it is well known, that even in more common pediatric clinical situations, such as urinary tract infections, these acknowledged existing guidelines will not always be followed [7]. Regarding underlying complex anatomical conditions the physician has to be aware of the limited availability of appropriate instruments and difficulties due to surgical positioning in complex associated muscular or skeletal deformities. These anatomical challenges might as well have an effect on the chosen course of treatment as other variables such as stone characters or the experience of the treating surgeon.

Five of our 24 patients underwent a low dose CT-scan thereby generating HU values of their respective stones. Four of these patients were treated according to guidelines. In this preliminary study stone characters and especially their composition did not seem to have an influence on the chosen treatment pathways. It has to be mentioned though, that stone analysis was only possible in a small proportion of the included patients. So, in this respect further investigations in a larger cohort are necessary.

Concerning the experience of surgeons, both institutions regularly treat a high volume of adult stone patients. In 2017 171 ureterorenoscopies (URS), 40 percutaneous treatments (PCNL), and 101 shock-wave lithotripsies (SWL) were carried out in Stuttgart, 283 URS, 48 PCNL and 143 SWL in Ulm respectively. It could be shown in literature [8] that retrograde intrarenal surgery performed by an expert adult surgeon in a pediatric population is a safe option. As pediatric stone cases are rare but a high volume of adult patients are treated by our experts every year, all possible treatment options could be offered to our patients in both centers with suitable expertise. However, we do insist upon an interdisciplinary treatment of pediatric patients on a pediatric ward, if necessary, and together with a pediatric nephrologists.

Reviewing the available literature showed a paucity of papers investigating the extent of guideline-conform clinical approaches in stone patients, especially with a focus on pediatric patients.

For adults, Wendt-Nordahl et al. published a prospective trial with 30 adult cases (21 male, 9 female) of symptomatic urolithiasis in 2008 [9]. 23 of their 30 patients (76.7%) were treated adhering to the current EAU guidelines of 2007. Three of these seven not guideline-conform treated adults insisted on this specific guideline-not-adherent treatment as a result of the informed consent concept. In the other four cases anatomical and medical conditions gave reasons for a divergent individual decision. 60% of their patients (18 of 30) needed a second or additional treatment, 61% (14/23) of the guideline-compliant and 57% (4/7) of the not-adherently treated patients, respectively [9]. In this current pediatric cohort 71% of the children were treated adherently to national and international guidelines. As mentioned above, in two cases parents refused consent to the recommended guideline treatment and the children were treated conservatively. In the other five individual approaches anatomical and medical conditions lead to guideline deviations. Consecutively, there was no significant difference in regards of necessary secondary or adjunctive treatments between the guideline-adherently and guideline-non-adherently treated children (*p* = 1.0). As described in literature before [10, 11] and due to the assumed less invasive treatment method, children undergoing SWL treatment needed repeated and adjunctive treatment in 80% of the cases. As children need anesthesia for that intervention, this fact should clearly be considered and addressed during initial parental discussion. Interestingly, six of seven conservatively treated children (86%) did not need further treatment to successfully pass ureteral stones of a size of up to 7 mm (median 4.1 mm). This reflects the fact that pediatric ureters show a higher degree of flexibility and larger transport capacity than adult ureters [3, 4, 12]. In this context, Mokhless et al. conducted a prospective randomized controlled trial in children, median 8 years of age, with distal ureteric calculi < 12 mm, that demonstrated a significant higher stone free rate and a shorter mean stone expulsion time with the use of oral tamsulosin [13]. In our current pediatric cohort, five of seven (median age 16.2 years) of the above mentioned conservatively treated children received tamsulosin. Four children passed their stones spontaneously. One boy however needed a subsequent URS for complete stone removal. In general, only one third of potential pediatric candidates for MET receive this safe and effective but yet off-label-use therapy, so further standardized protocols and management recommendation are needed [14]. In Summary, our current data seem to support the experience of Wendt-Nordahl et al. in adults [9]. Guideline-adherence in a pediatric stone setting was similar to those in an adult cohort [9]. Nevertheless, guideline recommendations did not match the needs or wishes of up to 30% of the stone patients in both trials. Furthermore, guideline-non-adherent treatment did not necessarily cause a higher rate of secondary or adjunctive treatments or cause a higher rate of morbidity. Although not significant, in the current pediatric cohort the rate for secondary or adjunctive treatments tend to be slightly lower in the guideline-not-conform treated children. However, due to this small population and preliminary design further investigation of a larger cohort is needed to support this assumption.

This study has several limitations. According to the fact that data retrieval was retrospective, missing or inconsistent data did occur. As patient numbers were small the question whether pediatric guideline recommendations were clinically feasible or not cannot unerringly be extrapolated onto a larger cohort. We therefore plan to conduct a German multicenter study to further evaluate this topic. The collected data nevertheless show similar results regarding guideline-conform clinical approach as those in adults. However, a prospective trial is needed to verify the clinical feasibility of the current pediatric guideline recommendations.

## Conclusion

Due to the sparse systematic literature pediatric guidelines in stone disease are mainly based on editorial systematic literature review condensed with personal experience of the guideline authors. Therefore, these clinically derived guidelines might not necessarily cover all aspects of daily pediatric urological routine work sufficiently. Nevertheless the current 2015 German s2k-guidelines and the 2014 EAU guidelines on pediatric urolithiasis matched in more than 70% of the unselected pediatric cases in two institutions in southwestern Germany. The cases of guideline-non-adherence in general show the strength but also the weakness and limitations of medical guidelines. Neither the guideline-adherently treated nor the not-adherently treated children needed more secondary or adjunctive treatments. However the 30% deviation rate demonstrates that individual clinical conditions may also need an individually tailored therapy approach, especially for pediatric patients.

## Data Availability

The datasets used and/or analyzed during the current study are available from the corresponding author on reasonable request.

## Abbreviations

MET: Medical expulsion therapy
URS: Ureterorenoscopy
DJ: Double-J ureteral stent
SWL: Shock-wave lithotripsy
PCNL: Percutaneous nephrolithopraxy
